# Comparing hemoglobin distributions between population-based surveys matched by country and time

**DOI:** 10.1186/s12889-020-08537-4

**Published:** 2020-03-30

**Authors:** Daniel J. Hruschka, Anne M. Williams, Zuguo Mei, Eva Leidman, Parminder S. Suchdev, Melissa F. Young, Sorrel Namaste

**Affiliations:** 1 School of Human Evolution and Social Change, Arizona State University, Tempe, AZ USA; 2 Centers for Disease Control and Prevention, Atlanta, GA USA; 3 McKing Consulting Corporation, Atlanta, GA USA; 4 Department of Pediatrics, Emory University School of Medicine, Atlanta, GA USA; 5 Rollins School of Public Health, Emory University, Atlanta, GA USA; 6 The DHS Program, ICF International, Rockville, MD USA

**Keywords:** Anemia, Biomarkers reflecting inflammation and nutritional determinants of Anemia, Blood collection, Data quality, Demographic and health surveys, Hemoglobin, Micronutrient surveys, Nutrition surveys

## Abstract

**Background:**

Valid measurement of hemoglobin is important for tracking and targeting interventions. This study compares hemoglobin distributions between surveys matched by country and time from The Demographic and Health Survey (DHS) Program and the Biomarkers Reflecting Inflammation and Nutritional Determinants of Anemia (BRINDA) project.

**Methods:**

Four pairs of nationally representative surveys measuring hemoglobin using HemoCue® with capillary (DHS) or venous (BRINDA) blood were matched by country and time. Data included 17,719 children (6–59 months) and 21,594 non-pregnant women (15–49 y). Across paired surveys, we compared distributional statistics and anemia prevalence.

**Results:**

Surveys from three of the four countries showed substantial differences in anemia estimates (9 to 31 percentage point differences) which were consistently lower in BRINDA compared to DHS (2 to 31 points for children, 1 to 16 points for women).

**Conclusion:**

We identify substantial differences in anemia estimates from surveys of similar populations. Further work is needed to identify the cause of these differences to improve the robustness of anemia estimates for comparing populations and tracking improvements over time.

## Background

Anemia is a widespread public health problem associated with morbidity and mortality among women and children [1–3]. For this reason, high quality anemia data from population-based surveys are essential for monitoring progress toward meeting global health goals and advocating for appropriate action in populations at greatest risk. Defined as a hemoglobin concentration below a certain threshold, anemia is caused by factors that affect the morphology, production, turnover, loss, or destruction of red blood cells [1]. The World Health Organization (WHO) recommends the use of hemoglobin concentrations to estimate the population prevalence of anemia [4]. Thus, precise, accurate, and reliable measurement of hemoglobin concentrations is critical for generating valid and robust anemia estimates and informing the prevention and control of anemia.

Many factors can influence population-based anemia estimates. These include environmental factors that differentially affect accuracy of each method (e.g., humidity) [5], the use of venous or capillary specimens [6], the instrument used to assess hemoglobin [7], season of measurement [8–10], iron supplementation [6], and biological variability, such as prevalence of alpha thalassemia [6, 11, 12]. For population-based surveys, the HemoCue® analyzer is the most routinely used device to measure hemoglobin concentrations in field settings (HemoCue AB, Angelholm, Sweden) system [4]. This device is relatively inexpensive, easily portable, does not require a cold chain, and produces a result within a minute. It is generally considered to be capable of providing precise and accurate measurements of hemoglobin concentrations using either capillary blood—obtained by puncturing the ball of the finger or heel—or venous blood—obtained by direct puncture to a vein [13, 14]. However, emerging evidence indicates many methodological factors, including pre-analytic factors [15], can influence hemoglobin readings using the HemoCue®. Variance in the collection of capillary blood can lead to falsely low readings and in some cases higher readings [7, 16, 17]. Incorrect techniques include not allowing alcohol solution to dry before puncturing the finger, shallow puncture, squeezing or milking the finger, microcuvette contains air bubbles, incomplete filling and re-dipping of microcuvette, damaged or expired microcuvettes and blood clotting [17]. Apart from the blood collection technique, studies have cited inherent drop-to-drop variability in hemoglobin concentrations [18].

Compared to research on anthropometric data quality in population-based surveys [19–21], research aimed at assessing the robustness of hemoglobin and anemia estimates in population-based surveys is still in its infancy [6, 22]. Given the importance of international population-based anemia estimates for tracking progress toward global health goals and prioritizing action for the most vulnerable populations, there is a need for work exploring the stability and replicability of these estimates from nationally-representative surveys. For this study, we identified pairs of nationally-representative surveys collected from the same country within relatively short periods of time (0 to 2 years) and compared hemoglobin concentration distributions (e.g., means, standard deviations, skewness and kurtosis) and estimates of anemia prevalence between these surveys. The rationale for this is that anemia estimates from such surveys would have a high likelihood of being used interchangeably to derive estimates for a country close to that point in time [2]. Paired surveys were selected from two sources of population-based survey data in low- and middle-income countries (i.e., the BRINDA project and The DHS Program). Our objective was to identify potential differences between these population-based surveys in these key indicators.

## Methods

### Data

The Demographic and Health Surveys (DHS) Program is one of the principal sources of global data on hemoglobin and uses capillary blood to assess hemoglobin concentrations with the HemoCue® device. The second set of surveys came from the BRINDA project—a database composed of harmonized micronutrient surveys. This database was created as part of a multi-agency collaboration to address programmatic issues related to inflammation, micronutrient biomarkers, and anemia etiology [23–25]. Like surveys from The DHS Program, the existing set of surveys in the BRINDA project database includes low- and middle-income countries from a range of world regions, including sub-Saharan Africa, South Asia, and Central Asia. The surveys in the BRINDA project database considered here used venous blood to assess hemoglobin concentrations using the HemoCue® device.

To control for population characteristics, we compared different nationally representative surveys from the BRINDA project and The DHS Program in the same country within two years of each other. Both platforms sample households from the official, national sampling frame. As such, while samples from survey pairs were independent, the target populations were approximately the same. One potentially crucial distinction across all survey pairs was the use of venous versus capillary blood collection, with BRINDA project surveys using venous and surveys from The DHS Program using capillary blood.

The DHS Program collects data on anemia through two kinds of surveys—Demographic and Health Surveys (DHS) and Malaria Indicator Surveys (MIS). For each nationally representative survey from The DHS Program with hemoglobin data, we searched for a nationally representative survey in the BRINDA project database from the same country which was within two years of the DHS survey. When more than one eligible DHS or MIS survey was matched to a single survey in the BRINDA project database, we chose the DHS or MIS survey with interview dates that were closest to the interview dates of the survey in the BRINDA project database. Two BRINDA project datasets were derived from the same data as the corresponding DHS survey, and so these were excluded from analyses. This selection process resulted in four pairs of surveys conducted between 2009 and 2016 from four countries in sub-Saharan Africa and South Asia.

All surveys were based on cluster-based sampling designed to provide nationally representative population estimates. In the DHS surveys used in this analysis, hemoglobin concentrations were assessed either in all households or in a randomly selected subpopulation of households. In half of all sampled households in one DHS survey (Cameroon), and one-third of households in two DHS surveys (Bangladesh and Malawi) the hemoglobin measurements were taken on all children (6–50 months) and reproductive age women (15–49 years) present in the households. In the only MIS survey included in these analyses (Liberia), all sampled households were selected, and hemoglobin was collected from all children, but not reproductive age women, in the selected households (Table S1).

Among the surveys in the BRINDA project database, all sampled households in Liberia and Cameroon were selected, whereas 20% of sampled households were selected in Bangladesh for hemoglobin measurement. In Malawi, all sampled households were selected for hemoglobin data collection for children (6–59 months) but only 45% of sampled households were selected for reproductive age women (15–49 years). Within selected households in Cameroon, one eligible child (12–59 months) and one eligible woman (15–49 years) were randomly selected. In Bangladesh, Liberia and Malawi, all eligible individuals within selected households were selected [children (6–59 months), reproductive age women (15–49 years), except in Liberia children (6–35 months)].

The Malawi 2015–2016 survey in the BRINDA project database was conducted in conjunction with the Malawi 2015–2016 DHS survey and was collected from a sub-sample of the households selected for the DHS survey.

In both The DHS Program and BRINDA project surveys analyzed here, hemoglobin concentrations were assessed with a portable hemoglobinometer. The type of device used in the four surveys included here is unknown; during the time period of these surveys the DHS and MIS surveys typically used the Hemocue 201+ but did occasionally use Hemocue 301. All surveys in the BRINDA project database used HemoCue 201+, except Malawi which used HemoCue 301. In the DHS and MIS surveys, the protocol was to obtain blood from heel pricks for children 6–11 months and finger pricks for individuals 12 months or older [22]. The DHS and MIS final reports did not specify the blood drop used, but the typical procedure has been to use the third drop of blood. The exception is when the assessment of hemoglobin concentrations has been combined with malaria or HIV, in which case the fourth and fifth drops of blood have been used, respectively [22]. In the surveys in this analysis, malaria was measured in Liberia and Cameroon in children, and HIV was measured in countries Cameroon and Malawi in women. The four BRINDA project surveys used a drop of blood from the vacutainer (pooled sample).

#### Exclusions

Consistent with prior analyses of surveys from The DHS Program, we included only usual or de jure residents in analyses to ensure that altitude measurements were relevant to the participants’ living conditions [22]. Such information was not available for BRINDA project surveys. A number of exclusions were also made to increase comparability between the BRINDA project and The DHS Program. First, we restricted analyses to cases that had values for age, sex, and hemoglobin. We consider children of age 6–59 months. Due to restricted samples in the surveys in the BRINDA project database, the Cameroon analysis considered children 12–59 months and the Liberia analysis considered children 6–35 months. We excluded women who reported being pregnant. Based on guidelines proposed by previous researchers [26], we excluded adjusted hemoglobin concentrations outside of the following ranges—4.0 g/dL to 18.0 g/dL for women and children [22, 26].

#### Adjusting for altitude and smokin

Only some of the surveys in the BRINDA project database included data for altitude. To ensure comparability between surveys from the same country while also adjusting for altitude when possible, we adjust for altitude only in countries when both surveys for that country include values for it (Malawi). Two other countries were uniformly below 1000 m altitude and thus required no adjustment (Bangladesh and Liberia). Altitude was missing from the Cameroon survey in the BRINDA project database and therefore no adjustment was made; however, altitude may have been relevant in this case.

There were no BRINDA-DHS country pairs where both surveys included information on smoking. In those countries where smoking was recorded, it was very rare (< 1% of women in countries Cameroon and Malawi). In the two countries without smoking data for either BRINDA project or The DHS Program (Bangladesh and Liberia), the World Bank estimates that fewer than 3% of women smoke [27]. Given the negligible prevalence of smoking among women in all BRINDA-DHS country pairs, we do not adjust to maintain comparability between surveys from the same country.

### Key variables

For the primary analyses, we examine distributional properties of hemoglobin, including dispersion, skewness and kurtosis, and estimates of any, mild, moderate, and severe anemia. We also report survey-specific digit preference in supplemental materials (Table S1).

#### Distributions of hemoglobin concentrations

To examine differences in the distributions of hemoglobin between samples from the same country, we estimated the standard deviation, skewness, and kurtosis of the distributions of concentrations. To our knowledge, there are no systematically validated guidelines for dispersions of hemoglobin concentrations that reflect acceptable data quality. However, a commonly cited review article proposed that “surveys or surveillance systems with apparently acceptable quality technique using HemoCue© tend to be in the 1.1-1.5 range”, an observation based on the authors’ empirical experience [26]. Based on this guidance, we flagged standard deviations that lie outside of 1.1 to 1.5 as potentially worth investigation [26]. We report these because they may be of interest to researchers, but using this range to identify acceptable quality technique is premature pending more systematic study.

#### Anemia

According to WHO guidelines, we defined different levels of anemia based on adjusted hemoglobin concentrations (in g/dL) and the following cutoffs. Children 6–59 month: any anemia < 11.0, mild 10.0–10.9, moderate 7.0–9.9, severe < 7.0. Non-pregnant women 15–49 year: any anemia < 12.0, mild 11.0–11.9, moderate 8.0–10.9, severe < 8.0 [4, 22].

#### Contextual variables

When available, we also report survey-level variables—months in which survey was conducted and prevalence of iron supplementation and malaria among children—that may be relevant for anemia estimates (Table 2). Reported iron supplementation among children in the last seven days was collected for DHS surveys in Bangladesh and Malawi and BRINDA project surveys in Bangladesh and Cameroon. In the Malawi survey in the BRINDA project, iron supplementation was reported in the last month. Children were tested for malaria with: (1) rapid diagnostic tests in DHS surveys in Liberia and Cameroon and BRINDA project surveys in Liberia and Malawi, and (2) blood smears in the DHS survey from Liberia and the BRINDA project survey from Cameroon. Population estimates are reported for children ages 6–59 months children in Bangladesh and Malawi, 6–35 months in Liberia, and 12–59 months in Cameroon.

### Analyses

For each pair of surveys, we used unweighted and non-design effect analysis for distribution estimates (e.g., standard deviations, skewness, kurtosis), to focus on the variance of individual measurements and not population-level estimates. Mean adjusted hemoglobin and the prevalence of anemia classified as any, mild, moderate or severe was adjusted for survey design to compare nationally representative estimates between surveys. Analyses in Additional file 1: (Table S2) show that estimates unadjusted and adjusted for survey design were highly correlated (of 20 indicators, 15 with r > 0.90 and all but one with r > 0.80; child skewness). We also reported mean hemoglobin (Additional file 1: Figure S2) and prevalence of anemia (Fig. 3) by age (6–11.9 months, 12–23.9 months, 24–35.9 months, and 36–59 months for children; 15–19.9 years, 20–29.9 years, 30–39.9 years, 40–49.9 years for women).

To assess any potential differences between paired surveys, we used tests adjusted for survey design to ensure inferences are based on appropriate standard errors. We used a design-based F-test for comparing proportions for dichotomous variables (implausible values, any, mild, moderate, and severe anemia), and t-tests of mean difference for continuous variables (adjusted hemoglobin concentrations). We used the delta method to test differences in variance, skewness, and kurtosis [28]. To test whether distributions of hemoglobin concentrations deviate from normality, we used a Shapiro-Wilk test as an overall test of normality as well as specific tests of skewness and kurtosis [29]. Given the large number of tests of differences between paired surveys and of deviations from normality, we set alpha at 0.001 for these tests. To examine the correlation between mean and standard deviation of adjusted hemoglobin across all surveys, we used a Pearson correlation coefficient.

## Results

### Summary of samples

The combined sample of The DHS Program and BRINDA surveys with no missing values for hemoglobin and age included 17,719 children (13,669 DHS, 4050 BRINDA) and 21,594 non-pregnant reproductive age women (19,016 DHS, 2578 BRINDA, 15–49 year) (Table 1). For countries with available data from both surveys, malaria prevalence (Liberia and Cameroon), and recent iron supplementation among children (Bangladesh) was roughly comparable between paired surveys (Table 2).

**Table 1:** Survey characteristics

	Type	Year	Child N	Women N	Blood collection	Hb analysis	Drop #^a^
Bangladesh	DHS	2011	2241	4982	Capillary	201 + ^b^	3 C, 3 W
	BRINDA	2012	607	1033	Venous	201+	Pooled
Liberia	DHS	2011	1677	NA	Capillary	201 + ^b^	4 C
	BRINDA	2011	1456	NA	Venous	201+	Pooled
Cameroon	DHS	2011	4523	6768	Capillary	201 + ^b^	3 C, 5 W
	BRINDA	2009	815	775	Venous	201+	Pooled
Malawi	DHS	2015–2016	5228	7266	Capillary	201 + ^b^	3 C, 5 W
	BRINDA	2015–2016	1172	770	Venous	301	Pooled

*BRINDA* Biomarkers Reflecting Inflammation and Nutritional Determinants of Anemia project. *C* child, *DHS* The Demographic and Health Survey Program, *Hb* hemoglobin, *NA* Not Available, *W* women, ^a^Drop # is based on the standard procedures in The DHS Program-7 at the time this manuscript was published and not individual survey reports. ^b^The DHS and MIS surveys typically used HemoCue 201+ and in a few instances the 301, but this is not documented

**Table 2:** Survey differences child iron supplementation and childhood malaria prevalence

Country	Type	Season of survey (Start month-end month)	Ages (months)	Child iron supplementation^a^	Childhood malaria prevalence
Bangladesh	DHS	July-Dec	6–59	2%	NA
	BRINDA	Oct-Dec	6–59	2%	NA
Liberia	DHS	Nov-Dec	6–35	NA	22% ^b^,38% ^c^
	BRINDA	Apr-June	6–35	NA	30% ^c^
Cameroon	DHS	Jan-Aug	12–59	NA	32% ^c^
	BRINDA	Oct-Dec	12–59	1%	26% ^b^
Malawi	DHS	Oct-Feb	6–59	12%	NA
	BRINDA	Dec-Feb	6–59	3%	28%^c^

a. % of children given iron supplements in last 7 days (last month in Malawi BRINDA)

b. blood smears

c. rapid diagnostic testing

*NA* not available.

### Distributions of adjusted hemoglobin concentrations

**Fig. 1:**
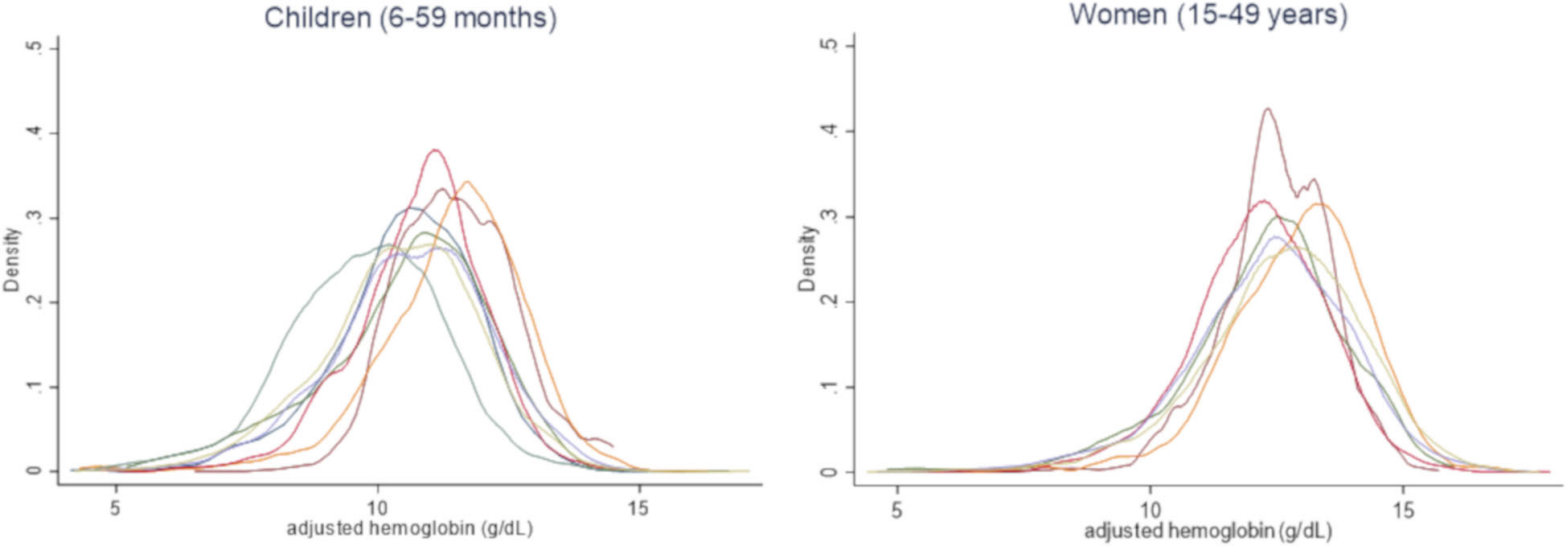
Distributions of hemoglobin concentrations for 8 children’s and 6 women’s surveys (adjusted for altitude). Kernel density plots. Data sources: seven pairs of country surveys conducted between 2009 and 2016 from the Demographic and Health Surveys (DHS) and the Biomarkers Reflecting Inflammation and Nutritional Determinants of Anemia (BRINDA). Total sample: 17,719 children and 21,594 women

Figure 1 illustrates the range of hemoglobin concentration distributions across the surveys for children (*n* = 8) and women (*n* = 6). All of the distributions were negatively skewed (mean child skewness = − 0.52, range = (− 0.99, − 0.23); mean women skewness = − 0.68, range = (− 0.39,-0.96)) and heavy-tailed (mean child kurtosis = 3.9, range = (3.2,5.7); mean women kurtosis = 4.6, range = (3.6,5.7)). Six of the fourteen surveys had hemoglobin distributions that fell outside of previously described bounds based on surveys with “acceptable quality technique using the HemoCue® system” [26]. In two of 8 child surveys, hemoglobin dispersions were greater than 1.5 standard deviation, and one survey—Bangladesh BRINDA—was less than 1.1 standard deviation [26]. In three of the 6 women’s surveys, hemoglobin dispersions were greater than 1.5 standard deviation (Tables 3 and 4). Distributions with dispersions that fell outside of these bounds did not differ between the BRINDA project and The DHS Program surveys.

**Table 3:** Properties of adjusted hemoglobin distributions among children (6–59 m)

country	survey	N	mean	SD	skew	kurtosis	% anemia			
							any	mild	mod	severe
Bangladesh	DHS-2011	2241	10.81^a^	1.23	−0.48	3.84	51^a^	29	21^a^	1^a^
	BR-2012	607	11.52	1.08	−0.58	4.13	33	28	5	0
Liberia	DHS-2011	1677	9.76^a^	1.49	− 0.23	3.19	79^a^	26	49^a^	4^a^
	BR-2011	1456	10.55	1.30	−0.52	3.75	59	31	28	1
Cameroon	DHS-2011	4523	10.63	1.54	−0.39	3.32	56	26	29	1
	BR-2009	815	10.54	1.62	−0.62	3.54	54	24	27	3
Malawi	DHS-2015/16	5228	10.44^a^	1.48	−0.34	3.43	61^a^	26^a^	33^a^	2
	BR-2015/16	1172	11.44	1.39	−0.99	5.69	30	18	11	1

^a^ within-pair difference significant at alpha = 0.001. Prevalence of 0 indicate prevalence less than 0.5%. Mean hemoglobin and % anemia estimates account for complex survey design. *SD* skew and kurtosis do not. All tests of between-survey differences are based on complex survey design. *BR* BRINDA, Biomarkers Reflecting Inflammation and Nutritional Determinants of Anemia project. DHS, The Demographic and Health Survey Program. any anemia < 11.0, mild 10.0–10.9, moderate 7.0–9.9, severe < 7.0. Hemoglobin concentrations adjusted for altitude

**Table 4:** Properties of adjusted hemoglobin distributions among women (15–49 y)

country	survey	N	mean	SD	skew	kurtosis	% anemia			
							any	mild	moderate	severe
Bangladesh	DHS 2011	4982	12.14^a^	1.36^a^	−0.39	3.80	42^a^	25	17	0
	BR 2012	1033	12.49	1.13	−0.69	5.72	26	18	8	0
Cameroon	DHS 2011	6768	12.33	1.66	−0.53	3.90	37	19	16	1
	BR 2009	775	12.25	1.60	−0.94	5.16	36	19	16	1
Malawi	DHS 2015/2016	7266	12.60^a^	1.66^a^	−0.62	4.27	31^a^	16	13^a^	1
	BR 2015/2016	770	12.91	1.39	−0.73	5.06	22	15	6	1

^a^ within-pair difference significant at alpha = 0.001. Prevalence of 0 indicate prevalence less than 0.5%. Mean hemoglobin and % anemia estimates account for complex survey design. SD, skew and kurtosis do not. All tests of between-survey differences are based on complex survey design. *BRINDA* BR, Biomarkers Reflecting Inflammation and Nutritional Determinants of Anemia project. DHS, The Demographic and Health Survey Program. any anemia < 12.0, mild 11.0–11.9, moderate 8.0–10.9, severe < 8.0. Hemoglobin concentrations adjusted for altitude

The adjusted hemoglobin distributions from the six women’s surveys and eight children’s surveys all significantly deviated from normality (Shapiro-Wilk *p* < 0.001). Moreover, all of the women’s surveys had negative skew and kurtosis that significantly deviated from a normal distribution (p < 0.001). Six of the eight child distributions had significantly heavy-tailed kurtosis (p < 0.001). The exceptions were the DHS and the BRINDA Project surveys from Cameroon, and the MIS survey from Liberia.

### Differences between distributions and prevalence estimates

**Fig. 2:**
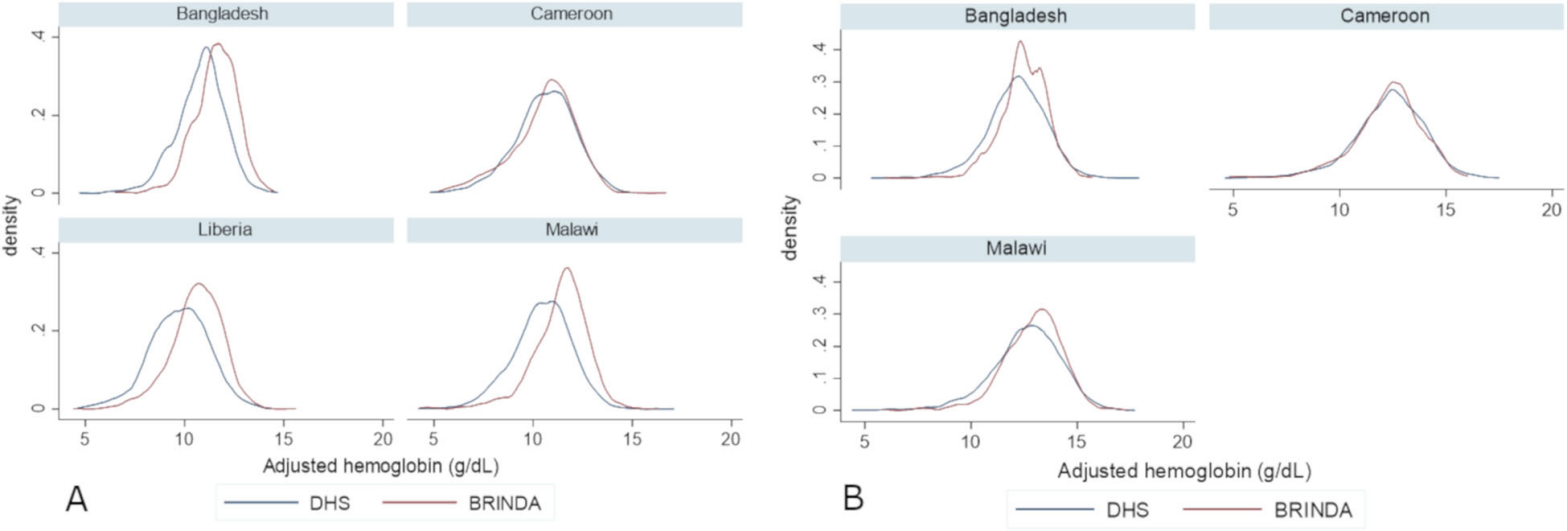
Hemoglobin distributions by country and survey type: **a** children, **b** women. Kernel density plots**.** Data sources: four pairs of country surveys conducted between 2009 and 2016 from the Demographic and Health Surveys (DHS) and the Biomarkers Reflecting Inflammation and Nutritional Determinants of Anemia (BRINDA). Hemoglobin concentrations adjusted for altitude

**Fig. 3:**
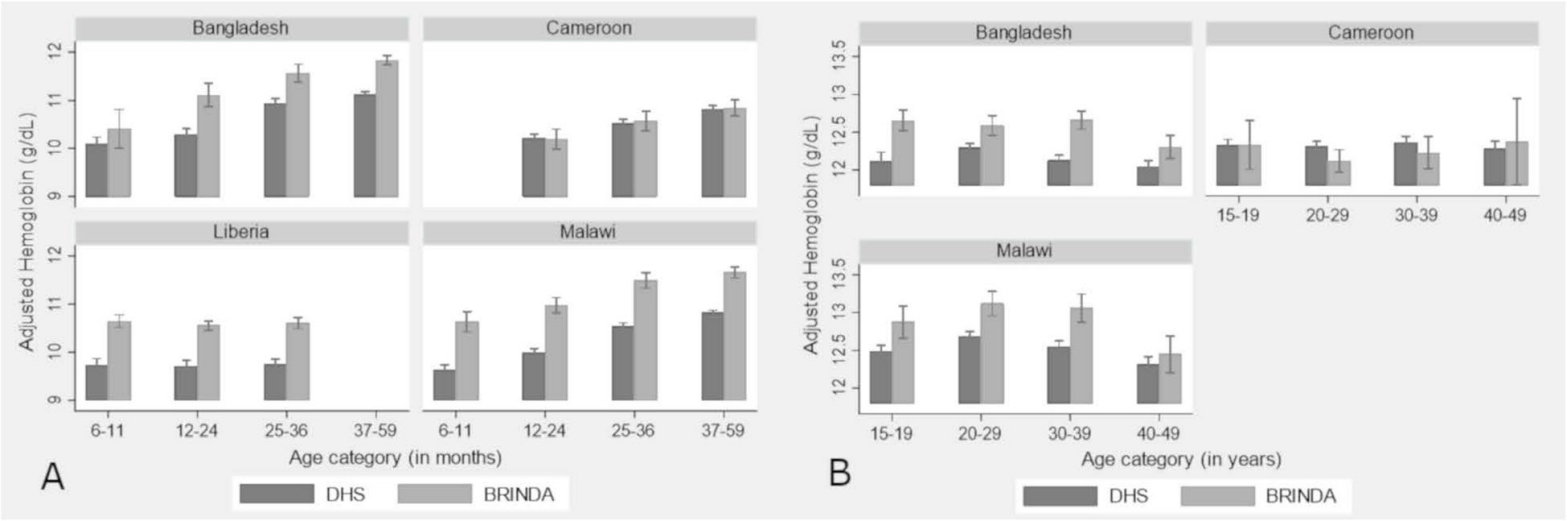
Proportion of any anemia by survey type and age for (**a**) children (6–59 months) and (**b**) women (15–49 years). Error bands are 95% CI. Data sources: six pairs of country surveys conducted between 2009 and 2016 from the Demographic and Health Surveys (DHS) and the Biomarkers Reflecting Inflammation and Nutritional Determinants of Anemia (BRINDA). Hemoglobin concentrations adjusted for altitude

Mean adjusted hemoglobin concentrations were significantly higher in BRINDA project child surveys in three countries (difference of 0.7 to 1.0 g/dL) and BRINDA project women’s surveys in two of the same countries (difference of 0.3 to 0.4 g/dL; Tables 3 and 4, Fig. 2). These three BRINDA project surveys also showed significantly lower prevalence of any anemia (18–31 percentage point difference in children, 9–16 percentage point difference in women) compared to the matched survey in The DHS Program (Table 5). Overall, the median difference in anemia prevalence between the BRINDA project and The DHS Program surveys was 19% for children and 9% for women. Fig. 3 illustrates these differences by age. At the same time, there was also substantial variation in the magnitude of these differences across countries, ranging from 2 percentage points in Cameroon to 31 percentage points in Malawi for children (Table 5).

**Table 5:** Percentage point difference in anemia estimates between DHS-BRINDA pairs

Country	Survey	Year	Sample size		Difference in any anemia % (DHS - BRINDA)	
			Children	Women	Children	Women
Bangladesh	DHS BRINDA	2011 2012	2241 607	4982 1033	**+ 18%**	**+ 16%**
Liberia	DHS BRINDA	2011 2011	1677 1456	NA	**+ 20%**	NA
Cameroon	DHS BRINDA	2011 2009	4523 815	6768 775	+ 2%	+ 1%
Malawi	DHS BRINDA	2015/2016 2015/2016	5228 1172	7266 770	**+ 31%**	**+ 9%**

Differences significant alpha = 0.001 in bold. *BRINDA* Biomarkers Reflecting Inflammation and Nutritional Determinants of Anemia project, *DHS* The Demographic and Health Survey Program. any anemia < 11.0 and < 12.0 among children and non-pregnant women respectively

Standard deviations were significantly different in one pair of child surveys and two pairs of women’s surveys, and in both cases the standard deviation was greater in the survey from The DHS Program. There were no significant differences in kurtosis or skewness between any of the paired surveys (Tables 3 and 4).

### Contextual differences between surveys

Two survey pairs—Bangladesh and Malawi—were collected during overlapping periods (Table 2), while two others—Liberia and Cameroon—were collected in non-overlapping periods. Notably, the two Malawi surveys were collected as part of a joint effort, with the micronutrient survey in the BRINDA project collected from a subsample of the DHS survey. Child iron supplementation was generally quite low in all surveys. In the two countries—Liberia and Cameroon—where both surveys tested children for malaria, the results were roughly comparable, though somewhat higher in the DHS surveys.

## Discussion

In this study, we compared hemoglobin distributions and anemia estimates between nationally representative surveys from two sources—The DHS Program and the BRINDA project database—matched by country, within two years of each other, and using a similar method for hemoglobin assessment, Hemocue©. Overall, we found marked differences in mean hemoglobin concentrations and anemia prevalence estimates between paired surveys in three of the four survey pairs, with consistently higher prevalence estimates in The DHS Program surveys (median percentage point difference for children = + 19%, for women + 9%). These differences are generally larger in the child surveys. The differences between paired surveys were also generally consistent across age categories (Fig. 3).

Given that some paired surveys were conducted in different seasons, it is possible that malarial seasonality may have played a role in these differences. Specifically, lower levels of anemia are typically found in the dry season and higher levels in the rainy season in places with high burden of malaria [8–10]. However, the paired surveys with the largest difference in anemia estimates (61% versus 30% in Malawi) were conducted within weeks of each other. Moreover, the other two surveys with available malaria data (Liberia and Cameroon) showed comparable prevalence of malaria among children. Another possible explanation for such differences in anemia estimates is variation in child iron intervention. However, the only country with data for both paired surveys (Bangladesh) on iron supplementation showed practically no iron supplementation (2% in both 2011 DHS and 2012 BRINDA) despite large differences in anemia estimates.

Differences in blood specimen type (venous or capillary) may also contribute to the observed differences in anemia prevalence [6]. However, if specimen type were the primary reason for differences in estimates, one would expect the percentage differences in prevalence to be similar across country contexts. Instead, substantial variability in difference between paired survey prevalence were observed (ranging from 1 to 31 percentage points). This variability in the difference between surveys using venous and capillary specimens is consistent with previous studies that have shown capillary blood can variously produce higher [30–32], lower [33], and comparable [5, 14] average hemoglobin concentrations when compared to venous blood [6, 34]. A recent paper found venous and capillary blood produced comparable results in a controlled environment, which points to the importance of good training to ensure the pre-analytical phase of data collection is done well [5]. A recent large-scale meta-analysis of hemoglobin measurement methods suggests that capillary fingerprick usually produces higher hemoglobin concentrations than venous blood—the opposite of our findings [35]. Others have found variability between blood drops even when collected by well-trained technicians in a controlled environment, which may indicate a pooled sample would reduce variability [18, 36]. Thus, there remain a number of unanswered questions about the direction and magnitude of differences in results based on the type and collection of blood specimen .

There may also be other as yet unmeasured factors that can lead to differences between surveys in anemia estimates. These may include the environmental factors that differentially affect accuracy of each method (e.g., humidity) [5], HemoCue® model [7, 37, 38], and biological variability [6, 11]. Differences in survey sampling procedures may also have played a role [39]. Surveys from The DHS Program selected all eligible participants, whereas in the surveys from the BRINDA project database, some selected only a subset of individuals within the household. Nevertheless, this is unlikely to solely account for the substantial differences seen between surveys. Another possible source of variation is that surveys from The DHS Program are implemented with the support and guidance of a single organization while micronutrient surveys included in the BRINDA project are carried out by varying organizations and are less harmonized. Further, the HemoCue© model was not consistent across surveys (e.g., 201+ versus 301). For example, in Malawi the DHS Program used the 201+ model, while the BRINDA survey used the 301.

While it is unlikely that any of these differences solely account for the substantial differences in estimates observed, the relative contribution of each is unknown, limiting the ability to interpret trends in estimates of anemia from national, population-representative surveys. At a minimum, these findings suggest that further study is necessary to understand the causes of such substantial variation. This will help determine what additional variables need to be recorded and reported to assist in contextualizing and interpreting hemoglobin estimates. These variables potentially include the specific details of blood specimen collection, HemoCue® model, and ambient humidity. A better understanding of how these and other nuisance variables influence hemoglobin estimates will assist researchers and policymakers interpret and compare estimates derived from diverse global sources.

Despite these unexpected differences in mean hemoglobin and anemia prevalence, we also found some common properties of hemoglobin distributions across all samples. First, hemoglobin distributions deviated significantly from a normal distribution, all with negative skew and heavy tails (i.e. kurtosis > 3). This is consistent with other studies of hemoglobin that have had sufficiently large samples to identify deviations from normality [22, 40, 41].

Notably, nearly half of the distributions (6 of 14 surveys) had standard deviations outside of the range noted in a previous review of cross-sectional surveys and surveillance systems (1.1 to 1.5) with apparently acceptable quality technique using the HemoCue© [26]. However, as noted earlier, these guidelines were based on authors’ empirical experience rather than a systematically designed validation study. Future systematic study of larger datasets and clear validation measures should assist in determining how properties of hemoglobin may indicate lower data quality and increased measurement error.

In these analyses, we made every effort to identify comparable surveys and to restrict samples to maximize comparability between surveys from the same countries. However, some critical differences deserve attention. First, we were unable to compare data completeness between survey type because there was insufficient information on refusal rates or other reasons for missing data among those eligible for hemoglobin testing for the surveys in the BRINDA database. Conceivably, if more caregivers of younger children refused testing, the prevalence of anemia could be artificially lower given younger children are at a greater risk of anemia [22]. Second, comparing surveys that were collected in the same year and where data collection covered similar months would be ideal for a more controlled comparison. Controlling by month may be particularly important in situations where disease burden and nutrition follow seasonal changes. The small number of survey pairs in this study also constrains our ability to examine potential reasons for those differences.

## Conclusion

By comparing nationally-representative surveys from the same country conducted within two or less years, we identified substantial differences in anemia estimates (median 16 percentage points (pp), range 1–31 pp) across all children’s and women’s surveys. Substantial differences in anemia estimates from population-based surveys conducted close in time can cause confusion for governments, program planners, and global anemia reduction trackers. Further work is needed to identify the cause of these different estimates, including potential effects of survey implementation or supervision, systematic method variance, and ecological conditions such as seasonal variation in disease burden and food insecurity.

## Supplementary information

**Additional file 1: Table S1.** Percentage of sampled households with hemoglobin measurements. **Table S2**. Correlations of unweighted and survey-weighted estimates of key variables. **Figure S2**. Mean hemoglobin concentration by country and survey type (a) children and (b) women.

## Data Availability

The DHS Program datasets analyzed during the current study are available in The DHS Program repository (https://dhsprogram.com/). The BRINDA project data is not publicly available but the project is working towards establishing a repository of micronutrient survey data (https://brinda-nutrition.org/). The data for the Malawi 2015–2016 micronutrient survey stored by the BRINDA project is also available in the DHS Program repository.

## Abbreviations

BRINDA: Biomarkers Reflecting Inflammation and Nutritional Determinants of Anemia
DHS: Demographic and Health Survey

## References

[1] Balarajan Y, Ramakrishnan U, Özaltin E, Shankar AH, Subramanian S (2011). Anaemia in low-income and middle-income countries. Lancet.

[2] Stevens GA, Finucane MM, De-Regil LM, Paciorek CJ, Flaxman SR, Branca F, Peña-Rosas JP, Bhutta ZA, Ezzati M, Group NIMS (2013). Global, regional, and national trends in haemoglobin concentration and prevalence of total and severe anaemia in children and pregnant and non-pregnant women for 1995–2011: a systematic analysis of population-representative data. Lancet Glob Health.

[3] Daru J, Zamora J, Fernández-Félix BM, Vogel J, Oladapo OT, Morisaki N, Tunçalp Ö, Torloni MR, Mittal S, Jayaratne K (2018). Risk of maternal mortality in women with severe anaemia during pregnancy and post partum: a multilevel analysis. Lancet Glob Health.

[4] World Health Organization (2011). Haemoglobin concentrations for the diagnosis of anaemia and assessment of severity.

[5] Whitehead RD, Zhang M, Sternberg MR, Schleicher RL, Drammeh B, Mapango C, Pfeiffer CM (2017). Effects of preanalytical factors on hemoglobin measurement: a comparison of two HemoCue® point-of-care analyzers. Clin Biochem.

[6] Neufeld LM, Larson LM, Kurpad A, Mburu S, Martorell R, Brown KH. Hemoglobin concentration and anemia diagnosis in venous and capillary blood: biological basis and policy implications. Ann NY Acad Sci. 2019;1450:172–89. 10.1111/nyas.14139 PMC7496102 31231815

[7] Rappaport AI, Barr SI, Green TJ, Karakochuk CD (2017). Variation in haemoglobin measurement across different HemoCue devices and device operators in rural Cambodia. J Clin Pathol.

[8] Hlimi T (2015). Association of anemia, pre-eclampsia and eclampsia with seasonality: a realist systematic review. Health Place.

[9] Roba KT, O'Connor TP, Belachew T, O'Brien NM (2015). Seasonal variation in nutritional status and anemia among lactating mothers in two agro-ecological zones of rural Ethiopia: a longitudinal study. Nutrition.

[10] Ardiet D-L, Graz B, Szeless T, Mauris A, Falquet J, Doumbo OK, Dolo A, Guindo O, Sissoko MS, Konaré M (2014). Patterns of malaria indices across three consecutive seasons in children in a highly endemic area of West Africa: a three times-repeated cross-sectional study. Malar J.

[11] Karakochuk CD, Rappaport AI, Barr SI, Green TJ. Mean hemoglobin concentrations in fasting venous and non-fasting capillary blood of Cambodian women using a hemoglobinometer and an automated hematology analyzer. Clin Chem Lab Med. 2017;55(11):e247–e250. 10.1515/cclm-2017-0118 28412720

[12] Muncie JH, Campbell J (2009). Alpha and beta thalassemia. Am Fam Physician.

[13] Cohen AR, Seidl-Friedman J (1988). HemoCue® system for hemoglobin measurement: evaluation in anemic and nonanemic children. Am J Clin Pathol.

[14] Sari M, Sd P, Martini E, Herman S, Bloem MW, Yip R (2001). Estimating the prevalence of anaemia: a comparison of three methods. Bull World Health Organ.

[15] Bonini P, Plebani M, Ceriotti F, Rubboli F (2002). Errors in laboratory medicine. Clin Chem.

[16] Karakochuk CD, Janmohamed A, Whitfield KC, Barr SI, Vercauteren SM, Kroeun H, Talukder A, McLean J, Green TJ (2015). Evaluation of two methods to measure hemoglobin concentration among women with genetic hemoglobin disorders in Cambodia: a method-comparison study. Clin Chim Acta.

[17] Burger SE, Pierre-Louis JN (2002). How to assess iron deficiency anemia and use the hemocue.

[18] Bond MM, Richards-Kortum RR (2015). Drop-to-drop variation in the cellular components of fingerprick blood: implications for point-of-care diagnostic development. Am J Clin Pathol.

[19] Mei Z, Grummer-Strawn LM (2007). Standard deviation of anthropometric Z-scores as a data quality assessment tool using the 2006 WHO growth standards: a cross country analysis. Bull World Health Organ.

[20] Assaf S, Kothari MT, Pullum T. An Assessment of the Quality of DHS Anthropometric Data, 2005-2014. DHS Methodological Reports No. 16. Rockville: ICF International; 2015.

[21] Corsi DJ, Perkins JM, Subramanian S (2017). Child anthropometry data quality from demographic and health surveys, multiple Indicator cluster surveys, and National Nutrition Surveys in the west Central Africa region: are we comparing apples and oranges?. Glob Health Action.

[22] Pullum TW, Collison DK, Namaste SM, Garrett D (2017). Hemoglobin Data in DHS Surveys: Intrinsic Variation and Measurement Error. DHS Methodological Reports.

[23] Namaste SM, Aaron GJ, Varadhan R, Peerson JM, Suchdev PS (2017). Methodologic approach for the Biomarkers Reflecting Inflammation and Nutritional Determinants of Anemia (BRINDA) project. Am J Clin Nutr.

[24] Merrill RD, Burke RM, Northrop-Clewes CA, Rayco-Solon P, Flores-Ayala R, Namaste SM, Serdula MK, Suchdev PS (2017). Factors associated with inflammation in preschool children and women of reproductive age: Biomarkers Reflecting Inflammation and Nutritional Determinants of Anemia (BRINDA) project. Am J Clin Nutr.

[25] Suchdev PS, Namaste SM, Aaron GJ, Raiten DJ, Brown KH, Flores-Ayala R, Group BW (2016). Overview of the biomarkers reflecting inflammation and nutritional determinants of Anemia (BRINDA) project. Adv Nutr.

[26] Sullivan KM, Mei Z, Grummer-Strawn L, Parvanta I (2008). Haemoglobin adjustments to define anaemia. Tropical Med Int Health.

[27] World Health Organization: Global health observatory (GHO) data. In*.*; 2015.

[28] Hosmer D, Lemeshow S, May S (2008). Appendix 1: the delta method. Applied Survival Analysis*:* Regression Modeling of Time-to-Event Data.

[29] Nestel P, Committee IS (2002). Adjusting hemoglobin values in program surveys. Washington, DC: International Nutritional Anaemia Consultative Group, ILSI Human Nutrition Institute.

[30] Patel AJ, Wesley R, Leitman SF, Bryant BJ (2013). Capillary versus venous haemoglobin determination in the assessment of healthy blood donors. Vox Sang.

[31] Neufeld L, García-Guerra A, Sánchez-Francia D, Newton-Sánchez O, Ramírez-Villalobos MD, Rivera-Dommarco J (2002). Hemoglobin measured by Hemocue and a reference method in venous and capillary blood: a validation study. Salud Publica Mex.

[32] Bahadur S, Jain S, Jain M (2010). Estimation of hemoglobin in blood donors: a comparative study using hemocue and cell counter. Transfus Apher Sci.

[33] Tong E, Murphy W, Kinsella A, Darragh E, Woods J, Murphy C, McSweeney E (2010). Capillary and venous haemoglobin levels in blood donors: a 42-month study of 36 258 paired samples. Vox Sang.

[34] Boghani S, Mei Z, Perry GS, Brittenham GM, Cogswell ME (2017). Accuracy of capillary hemoglobin measurements for the detection of anemia among US low-income toddlers and pregnant women. Nutrients.

[35] Whitehead RD, Mei Z, Mapango C, Jefferds MED (2019). Methods and analyzers for hemoglobin measurement in clinical laboratories and field settings. Ann N Y Acad Sci.

[36] Conway A, Hinchliffe R, Earland J, Anderson L (1998). Measurement of haemoglobin using single drops of skin puncture blood: is precision acceptable?. J Clin Pathol.

[37] Rappaport AI, Karakochuk CD, Whitfield KC, Kheang KM, Green TJ (2017). A method comparison study between two hemoglobinometer models (Hemocue Hb 301 and Hb 201+) to measure hemoglobin concentrations and estimate anemia prevalence among women in Preah Vihear, Cambodia. Int J Lab Hematol.

[38] Jain A, Chowdhury N, Jain S (2018). Intra-and inter-model reliability of Hemocue Hb 201+ and HemoCue Hb 301 devices. Asian J Transfus Sci.

[39] Gorstein J, Sullivan K, Parvanta I, Begin F. Indicators and methods for cross-sectional surveys of vitamin and mineral status of populations. Micronutrient Initiative (Ottawa) and the Centers for Disease Control and Prevention (Atlanta). 2007. p. 125–8.

[40] Tufts DA, Haas JD, Beard JL, Spielvogel H (1985). Distribution of hemoglobin and functional consequences of anemia in adult males at high altitude. Am J Clin Nutr.

[41] Khusun H, Yip R, Schultink W, Dillon DH (1999). World Health Organization hemoglobin cut-off points for the detection of anemia are valid for an Indonesian population. J Nutr.

