# Supplementary material for: Comparing hemoglobin distributions between population-based surveys matched by country and time
Source: BMC Public Health. 2020 Mar 30;20:422. doi: 10.1186/s12889-020-08537-4 (PMC7106890; doi:10.1186/s12889-020-08537-4)
Supplement: Supplementary file 1 — Additional file 1: Table S1. Percentage of sampled households with hemoglobin measurements. Table S2. Correlations of unweighted and survey-weighted estimates of key variables. Figure S2. Mean hemoglobin concentration by country and survey type (a) children and (b) women. [file 12889_2020_8537_MOESM1_ESM.docx]

**Table S.1. Percentage of sampled households with hemoglobin measurements.** When two percentages are present, the first is for children, and the second is for women.

| **Country** | **DHS/MIS** | **BRINDA** |
| --- | --- | --- |
| **Bangladesh** | 33% | 20% |
| **Liberia** | 100%/0% | 100% |
| **Cameroon** | 50% | 100% |
| **Malawi** | 33% | 100%/45% |

**Table S.2**. **Correlations of unweighted and survey-weighted estimates of key variables**

|  | **Children** | **Women** |
| --- | --- | --- |
| **% implausible** | 0.93 | 0.95 |
| **excess 0 & 5** | 0.87 | 0.87 |
| **Hb mean** | 1.00 | 0.97 |
| **sd** | 0.98 | 1.00 |
| **skewness** | 0.69 | 0.97 |
| **kurtosis** | 0.92 | 0.81 |
| **% any anemia** | 0.99 | 0.97 |
| **% mild** | 0.92 | 0.91 |
| **% moderate** | 1.00 | 0.98 |
| **% severe** | 0.98 | 0.89 |

Children: any anemia < 11.0, mild 10.0-10.9, moderate 7.0-9.9, severe < 7.0. non-pregnant women: any anemia < 12.0, mild 11.0-11.9, moderate 8.0-10.9, severe < 8.0. Hb, hemoglobin.

**Figure S.2. Mean hemoglobin concentration by country and survey type (a) children and (b) women.** Error bars are 95% CI.

BRINDA, Biomarkers Reflecting Inflammation and Nutritional Determinants of Anemia project. DHS, The Demographic and Health Survey Program. Hemoglobin concentrations adjusted for altitude, U.S. adjusted for smoking.
